# Management of post cholecystectomy Mirizzi’s syndrome

**DOI:** 10.4103/0972-9941.15244

**Published:** 2005-03

**Authors:** Simon Janes, L. Berry, B. Dijkstra

**Affiliations:** Department of General Surgery, Christchurch Public Hospital, Christchurch, New Zealand; * Department of Radiology, Christchurch Public Hospital, Christchurch, New Zealand

**Keywords:** Mirizzi syndrome, cystic duct remnant, retained calculus

## Abstract

Various strategies have been proposed for the management of retained calculi within the biliary tree following cholecystectomy. We present a unique case of a cystic duct remnant calculus causing Mirizzi syndrome, only the fourth such case of its kind. An open procedure was planned, however the calculus was eventually extracted endoscopically. The pathophysiology and management of Mirizzi syndrome and retained calculi within the cystic duct remnant are discussed along with the merits of a minimally invasive approach.

## INTRODUCTION

The incidence of retained or recurrent calculi in the biliary tree following cholecystectomy is between 1.1% and 7%.[1] Management depends on the location of the calculi within the biliary tree, and retained calculi in the cystic duct remnant are highly unusual. A cystic duct remnant calculus causing Mirizzi’s syndrome is exceedingly rare, with only 3 cases reported to date.[2–4] We present the management of a unique case of a cystic duct remnant calculus causing Mirizzi syndrome, only the fourth such case of its kind.

## CASE REPORT

A 36-year-old woman presented with a two-day history of epigastric pain, nausea and vomiting. One year previously she underwent laparoscopic cholecystectomy for recurrent biliary pain. Gall bladder histology demonstrated chronic inflammatory changes and fragmented gallstones, but no complete calculi.

Physical examination revealed she was afebrile but tender in the epigastrium, with no palpable masses. Liver function tests (LFTs) showed normal bilirubin and albumin, however liver enzymes were deranged (normal ranges): alkaline phosphatase 125 iU/L (30-120 iU/L), gamma glutamyl-transpeptidase (GGT) 138 iU/L (10-35 iU/L) and alanine aminotransferase (ALT) 154 iU/L (0-40 iU/L). Amylase, urea and electrolytes, full blood count, and coagulation studies were all normal. Magnetic resonance cholangio-pancreatography (MRCP) showed a normal common bile duct (CBD) with no evidence of calculi, however the cystic duct remnant was dilated distal to a round filling defect, suggesting a calculus within the remnant.

**Figure 1:**
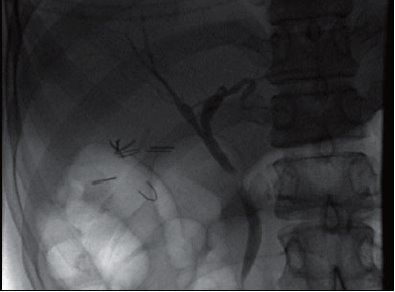
ERCP. The proximal CBD is narrowed and the lower segments of the right lobe of the liver and cystic duct remnant are not filled with contrast

Endoscopic retrograde cholangio-pancreatography (ERCP) demonstrated focal narrowing of the proximal CBD. The ducts of the left liver lobe and right upper lobe segments were patent but lower segments of the right lobe did not fill with contrast (Figure 1). Comparing the MRCP and ERCP suggested that the right lower lobe segments drained into the cystic duct remnant (an aberrant cholecystohepatic duct), which was occluded by a calculus compressing the CBD.

**Figure 2:**
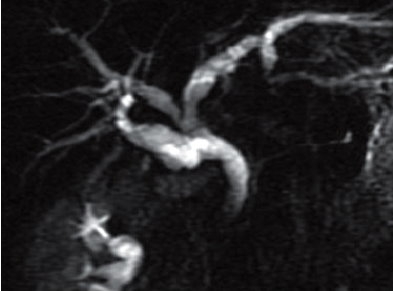
MRCP after stone migration. The cystic duct remnant remains dilated, with aberrant hepatic drainage clearly demonstrated

Elective laparotomy with exploration of the cystic duct remnant and CBD was planned; however, 24 hours later the patient developed worsening epigastric pain. She had developed icterus, with pale stools and dark urine. On examination she was jaundiced but her abdomen was non-tender. LFTs were consistent with biliary obstruction: bilirubin 134 umol/L (3-21 umol/L), ALP 281 iU/L, GGT 381 iU/L and ALT 435 iU/L. Repeat MRCP demonstrated CBD dilatation (10 mm) secondary to a calculus at its distal end (Figure 2). Presumably the calculus had migrated from the cystic duct remnant. The calculus was subsequently retrieved during ERCP. The patient made an uneventful recovery and has been symptom-free after 3 months follow-up.

## DISCUSSION

In 1948 Mirizzi described how calculi in the cystic duct or gall bladder infundibulum cause extrinsic compression of the common hepatic duct.[5] The eponymous syndrome has since been identified in 0.7-1.4% of all patients undergoing cholecystectomy.[6]

Gallstone impaction in Hartmann’s pouch or the cystic duct causes an inflammatory reaction, resulting in biliary obstruction. Calculi in a long parallel cystic duct predisposes to Mirizzi Type 1, where inflammation causes extrinsic bile duct compression. If inflammation persists, the gall bladder can adhere to the bile duct, causing pressure necrosis and fistula formation.[7] A cholecystobiliary fistula occluding one-third of the duct is a Mirizzi Type 2 abnormality, whereas occlusion of two-thirds of the duct or complete occlusion are classified as Mirizzi Type 3 and 4 respectively. ERCP is the primary method of diagnosing fistulae, and also has an important therapeutic role, including stone retrieval and stent placement.

An extensive review of 219 patients with Mirizzi syndrome showed that the vast majority of cases were Type 2 (41%) or Type 3 (44%), whereas Type 1 lesions occurred in only 11%.[7] Surgical management is controversial; however there is consensus that partial cholecystectomy and hepaticojejunostomy are the most appropriate procedures for Type 1 and 4 lesions respectively.[6,7] Various strategies have been used for Type 2 and 3 lesions, including percutaneous extraction, aiming to avoid bile duct stenosis. Whether fistulae closure is best achieved around a t-tube following cholecystectomy[6] or via choledochoplasty[7] is controversial, with most evidence coming from small case series.

Laparoscopic management of Mirizzi syndrome depends on the ability to delineate structures within Calot’s triangle, which can be difficult when a large impacted stone or fistula is present. Laparoscopic management of Type 1 cases is technically feasible and safe, whereas laparoscopic management of fistulae requires considerable skill and experience, and may be associated with significant morbidity and mortality due to retained CBD stones.

Three previous cases reports of Mirizzi syndrome due to cystic duct remnant calculi were successfully managed via open cholecystectomy[2,4] and endoscopically.[5] To our knowledge this is the first case of its occurrence in association with an anomalous cholecystohepatic duct— a rare anomaly. Although an anomalous cholecystohepatic duct may predispose to calculus formation, the short interval between cholecystectomy and readmission in this case (1 year) suggests that the calculus was retained rather than formed de novo. Furthermore, gall bladder histology demonstrated multiple gallstone fragments, which may have been present in the cystic duct remnant. Biliary anatomical variations are frequently encountered during cholecystectomy; awareness of these variations is a vital prerequisite before attempting biliary surgery.

## CONCLUSIONS

Retained cystic duct remnant stones are a rare cause of Mirizzi syndrome. Although it was fortunate in this case that endoscopic management was successful, cystic duct remnant calculi are probably best managed via an open procedure. In this case it was fortuitous that endoscopic retrieval was possible. Post-cholecystectomy Mirizzi syndrome should be considered as a cause of biliary obstruction once more common etiologies have been excluded.
